# Independent and Combined Relationships of Perceived Neighborhood Social Cohesion and Physical Frailty on Functional Disability in Community-Dwelling Older Adults

**DOI:** 10.3390/ijerph17165912

**Published:** 2020-08-14

**Authors:** Boqin Xie, Chenjuan Ma, Junqiao Wang

**Affiliations:** 1 School of Nursing, Fudan University, 305 Rd. Fenglin, Shanghai 200032, China; xieboqin@fudan.edu.cn; 2 Rory Meyers College of Nursing, New York University, New York, NY 10010, USA; cm4215@nyu.edu

**Keywords:** functional disability, physical frailty, perceived neighborhood social cohesion, healthy aging

## Abstract

Functional disability and physical frailty (PF) are debilitating geriatric conditions. Previous studies have suggested both perceived neighborhood social cohesion (PNSC) and PF can influence functional disability and may have an interactive effect too. This cross-sectional study aims to examine the independent and combined relationships of PF and PNSC on functional disability in community-dwelling older adults in Shanghai, China. A total of 1616 older adults aged ≥ 75 years were recruited using multistage sampling. Results showed that prefrailty, frailty (using the modified frailty phenotype criteria), and low PNSC (measured by the Neighborhood Cohesion Scale) were independently associated with increased likelihood of functional disability after adjustment of covariates. To evaluate the combined relationships of PF and PNSC, participants were classified into six groups based on their levels of PF and PNSC. The probability of frail older adults with low PNSC having functional disability stood out compared with the robust older adults with high PNSC. Our findings suggest the importance of high PNSC as a protective factor of maintaining functional ability. Future longitudinal studies are needed to identify the role of PNSC in the development of functional disability among frail older adults.

## 1. Introduction

Functional disability in older adults is a worldwide public health problem. It can be defined as limitations in the ability to perform daily activities that are essential to living an independent life [1]. Functional disability in older adults is associated with diminished quality of life, increased healthcare costs, and high rates of institutionalism and mortality [2,3,4,5]. China has the largest aging population in the world and the number of older adults aged 60 years or older is predicted to reach 402 million by 2040 [6]. With a rapid growth of aging population, the number of older adults with disabilities in China increases correspondingly. In 2013 alone, over 37 million older adults in China had significant declines in physical function. The number of Chinese older adults with functional disability is estimated to reach 66 million by 2050 [7,8]. The cost of long-term care associated with severe functional disability is estimated to reach 72.78 billion dollars by 2050 in China [9]. Given the enormous population and tremendous associated burden, prevention of disability is a priority in healthy aging in China as well as around the world. It is thus crucial to identify the factors associated with functional disability, especially those that are modifiable.

Physical frailty is a debilitating age-related syndrome of increased vulnerability and decreased physiological reserve capacity, which causes a wide range of adverse health outcomes [10,11]. An increasing number of studies has examined the associations between physical frailty and prevalence and incidence of functional disability [12,13,14,15]. It has been established that frail older adults are more likely to have functional disability compared with non-frail older adults. A growing body of evidence suggest neighborhood conditions and characteristics (e.g., neighborhood social cohesion) affect older adults’ health and well-being such as physical frailty [16,17,18,19]. Merging studies also indicate that, despite similar initial health conditions, older adults can develop very different health outcomes due to the neighborhoods and living environments [20,21].

Neighborhood social cohesion is considered as the perceived degree of connection among neighbors and people’s willingness to involve themselves for the common good [22]. It is also characterized by the levels of trust, norms of reciprocity and the formation of strong social bonds between the neighbors [23,24]. Neighborhood social cohesion has been measured at both community and individual level in research. Some researchers considered the community in its entirety and it was assumed to influence everybody in the neighborhood regardless of individual characteristics [23,25]. They therefore often conceptualized and operationalized neighborhood social cohesion at the aggregated level. In other studies, the researchers believed that a person’s perception of neighborhood boundaries may be different from the administrative boundary of a neighborhood he or she lives [18,24,26]. Therefore, forcibly aggregating the responses from people who do not consider themselves in the same neighborhood into a community-level indicator may lead to research bias. They thus often considered neighborhood social cohesion as an individual-level measure in their research.

Yet, regardless being conceptualized and measured at the community- or individual-level, merging evidence has demonstrated that better neighborhood social cohesion was associated with decreased risk of stroke [18] and myocardial infarction [17], mental health [23] and self-rated health [19,27,28]. When it comes to functional disability, one recent study has examined the impact of social cohesion on functional disability [29]. In this study, researchers measured aggregated-level social cohesion as the engagement in a particular social activity in past year among older adults and they reported a substantial association between high levels of social cohesion and low levels of functional disability. Clearly, the relationship between neighborhood social cohesion and functional disability needs to be better established.

At the same time, studies have suggested an interactive effect of social cohesion and physical frailty on functional disability [30,31]. Frail older adults with different levels of neighborhood social cohesion would have different risks of functional disability. However, the combined relationships of physical frailty and neighborhood social cohesion remain elusive. A better understanding of the individual and combined relationships of physical frailty and neighborhood social cohesion on physical disability can have the potential benefits of identifying older adults at high risk of functional disability. It also may contribute to developing effective interventions for preventing functional disability from an innovative perspective of the neighborhood or community.

The aims of this study were to examine the independent and combined relationships of physical frailty and perceived neighborhood social cohesion on functional disability in community-dwelling older adults in Shanghai, China. We hypothesized that: (1) physical frailty and low levels of neighborhood social cohesion would be independently associated with enhanced likelihood of functional disability; (2) a combination of physical frailty and low neighborhood social cohesion would substantially increase the likelihood of functional disability compared with physical frailty coexisting with high neighborhood social cohesion.

## 2. Methods

### 2.1. Participants

Data in this study were obtained from a parent study examining the prevalence of functional and cognitive disabilities among community-dwelling older adults in Shanghai. The parent study was conducted from June to August 2018 in Xuhui District, Shanghai, China. We conveniently selected three sub-districts from total of twelve sub-districts in Xuhui District, Shanghai, China. There are a total of eighty-eight neighborhoods in these three sub-districts. After approaching the neighborhood administrative officers, thirty-two neighborhoods were willing to participate in the parent study. Participants were then randomly selected among older adults aged 75 years or older using a community resident registry that included residents’ contacts in each community. We selected older adults aged 75 years or older as the sample in the parent study because the declines in functional and/or cognitive capabilities accelerate with advancing age and the parent study focused on the prevalent rates of functional and cognitive disabilities in middle-old and oldest-old older adults living in the community. The inclusion criteria of the parent study were older adults living in the community aged 75 years or older, without a clinical diagnosis of dementia, without a severe or unstable stage of somatic or psychiatric diseases, and willing to participate in the study. Power analysis was conducted to estimate the minimum sample size needed in the parent study. The sample size was calculated under the assumption that 18.8% of Chinese community-dwelling older adults aged 60 years or older would have disabilities in activities of daily living (ADL) based upon previous research [32]. With a margin of error of ±1% and a confidence level of 95%, a sample of at least 1467 participants was estimated. Initially, 2174 older adults were randomly selected in the parent study and 61 of them refused to participate. The parent study was approved by the Ethics Committee of the authors’ institute (Reference number: IRB#2017-12-03).

In the current study, 497 participants were excluded from the 2113 older adults in the parent study because they failed to complete the objective measurements of physical frailty (*n* = 439), the questionnaires of perceived social cohesion (*n* = 29) and/or ADL/IADL disability (*n* = 32). Ultimately, a total of 1616 community-dwelling older adults aged 75 or above were included in this study.

### 2.2. Data Collection Procedure

Data collection was performed and completed by a team of trained research assistants consisting of senior nursing undergraduate students. Potential participants were first contacted via phone by research assistants. After providing a brief overview of the study and their rights as participants, potential participants who expressed interest in and provided a verbal consent of participation were scheduled for a 45-min home visit to complete the study. Data were then collected by trained researchers at participants’ homes using a face-to-face interview modality. A written informed consent was obtained from all participants during the home visit before data collection.

### 2.3. Measures

#### 2.3.1. Functional Disability

Functional disability refers to limitations in the ability to perform basic and instrumental activities of daily activities (ADL/IADL) that are essential to living an independent life. The activities of the daily living scale were used to quantify functional disability. Participants were asked if they are able to perform the following tasks: 6 ADL tasks (dressing, eating, toileting, bathing, grooming, and transfers) and 8 IADL tasks (using the telephone, grocery shopping, preparing meals, housekeeping, laundry, driving or using public transportation, administering own medication, and handling money and goods) [33]. Each task was categorized into two levels: 0 = I can do it independently; 1 = I need partial or full assistance. The range of total score is from 0 to 14. The lowest total score of 0 indicates full independence without functional disability and a total score of 1 or above indicates functional disability.

#### 2.3.2. Physical Frailty

Physical frailty (PF) was measured using the modified CHS criteria of Fried et al. [10]. It includes five criteria, exhaustion, involuntary weight loss, weakness, slow walking speed, and low energy expenditure. Participants were considered frail with the presence of at least three of the five criteria, pre-frail with the presence of one or two criteria, and robust with none of them [10]. More specifically, exhaustion was determined by two questions that asked the older adults if they felt a lack of energy or were exhausted on more than 3 days during the past week. Involuntary weight loss was identified as a self-reported unintentional weight loss of 3 kg or more in the past six months. Weakness was assessed by grip strength of the dominant hand and using a dynamometer. Gender- and BMI-stratified cut-points as used in the CHS [10] were adopted in this study. A slow walking speed was considered as taking more than 14 s in the Time Up and Go test [34,35], in which participants were required to rise from a chair, walk three meters at their usual speed, turn around, walk back to the chair, and sit down. Energy expenditure was assessed using the duration of vigorous, moderate and light physical activities during a week, and low energy expenditure were identified using the gender-stratified lowest quartile of total physical activities duration [36].

#### 2.3.3. Perceived Neighborhood Social Cohesion

In this study, we focused on older adults’ perceptions of neighborhood social cohesion at the individual level and the term perceived neighborhood social cohesion (PNSC) was used. PNSC was assessed using an 8-item scale that was derived from the Neighborhood Cohesion Scale with good validity and reliability [23]. Using a 5-point Likert Scale from 1 = strongly disagree to 5 = strongly agree, the participants indicated the degree to which they agree with the following items: I visit my neighbors in their homes; the friendships and associations I have with other people in my neighborhood mean a lot to me; if I need advice about something I could go to someone in my neighborhood; I believe my neighbors would help in an emergency; I borrow things and exchange favors with my neighbors; I would be willing to work together with others on something to improve my neighborhood; I rarely have a neighbor over to my house to visit; I regularly stop and talk with people in my neighbors. The scores on each item were then summed (range from 8 to 40), and higher scores indicated higher perceived social cohesion. As suggested in the previous study [23], the scores were further dichotomized into two categories in this study: low PNSC ≤ 24 and high PNSC > 24. Cronbach’s alpha of the scale was 0.80 in this study.

#### 2.3.4. Covariates

Some factors associated with functional disability in the existing literature were considered as potential covariates in this study [37,38]. Socio-demographic data were collected, including age, gender, education level, marriage status, and household monthly income. A questionnaire regarding medical status was used to collect data on medical conditions (hypertension, stroke, heart diseases, diabetes mellitus, visional impairment, hearing impairment, urine incontinence and chronic pain), polypharmacy (currently taking ≥5 medications) [39], history of hospitalization and falls in the past year. Psychological data were collected, including depression and cognitive function. The Chinese 15-item version of the Geriatric Depression Scale (GDS-15) was used to identify depressive symptoms, and older adults with a GDS-15 score 5 or above were considered as having a depressive disorder. Cognitive function was assessed using the Chinese-version Mini-Mental Status Examination (MMSE-C) and the education-stratified cut-off points were used to identify cognitive impairment.

### 2.4. Data Analysis

Descriptive statistics were used to summarize the distribution of each study variable, including frequency and percentage for categorical variables, mean and standard deviation for continuous variables. Bivariate analyses were conducted to compare social-demographic, medical status, psychological factors, PF and PNSC between older adults with and without disabilities. Logistic regression analyses were used to evaluate the independent and combined impact of PF and PNSC on functional disability. Firstly, we tested if PF and PNSC were independently associated with functional disability in unadjusted and adjusted logistic models. Next, in order to examine the combined impact of PF and PNSC, we grouped participants into 6 groups based on their PF and PNSC, (1) robust with high PNSC; (2) robust with low PNSC; (3) pre-frail with high PNSC; (4) pre-frail with low PNSC; (5) frail with high PNSC; and (6) frail with low PNSC. A logistic model was used to estimate the likelihood of functional disability for each group when adjusting for sociodemographic, medical status, and psychological factors. Statistical significance was determined by a *p*-value < 0.05. With regard to subgroup analysis, our intention was to clarify the likelihood of functional disability for each subgroup compared with reference. All data management and analyses were performed using Stata 14 (Stata Corp, College Station, TX, USA).

## 3. Results

### 3.1. Sample Characteristics

The total sample consisted of 1616 community-dwelling older adults aged 75 years or older (mean age 81.44 ± 4.76; 58.7% female). Overall, nearly half of the participants (49.5%) had no more than 9 years education, and approximately one third of them were unmarried/single (single, divorced, or widowed) (34.3%) and had low family income (<5000 yuan per month, 33.7%). As shown in Table 1, the top five most common medical conditions in the sample were visional impairment (77.8%), hypertension (65.7%), hearing impairment (55%), chronic pain (48.8%) and heart diseases (39.1%). Cognitive impairment and depressive disorder accounted for 34.1% and 21.6%, respectively. Details of sample characteristics are shown in Table 1.

### 3.2. Rate of Functional Disability with Different Sample Characteristics

Overall, 23.76% of the older adults in this study had functional disability. As displayed in Table 1, adults with functional disability were more likely to be older, with less education (≤9 years), unmarried, low family income, with diagnosis of hypertension, stroke and heart diseases, with symptoms of urine incontinence and chronic pain, with impaired sensory function (visional/hearing), taking five or more medications, with history of hospitalization and/or falls in past year, with cognitive impairment and depressed (*p* < 0.05).

### 3.3. Physical Frailty and Perceived Neighborhood Social Cohesion According to Functional Disability

**Figure 1:**
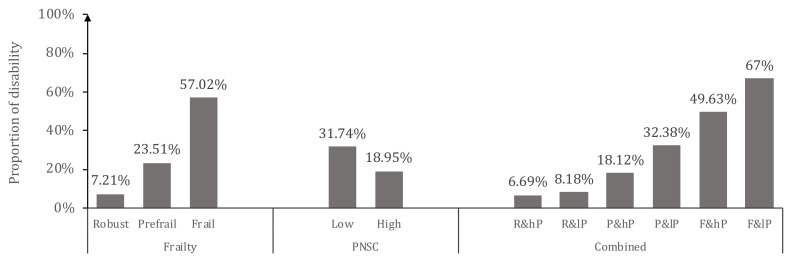
Proportion of functional disability by different status of physical frailty and/or perceived neighborhood social cohesion.Note: R = robust; P = Pre-frail; F = Frail; Hp = high level perceived neighborhood social cohesion (PNSC); lP = low level PNSC.

The rates of pre-frailty and frailty were 57.12% and 14.54% among all participants, respectively. The mean PNSC was 26.23 (SD = ± 5.18) and the percentage of low PNSC (PNSC score ≤24 out of a maximum score of 40) accounted for 37.6% in this study. Figure 1 shows the proportion of functional disability by different status of PF and/or PNSC. The percentage of older adults with functional disability ran from 7.2% in the robust group, 23.5% in the pre-fail group, to 57.0% in the frailty group (χ^2^ = 212.83, *p* < 0.001). There was a significant difference in the proportions of disability between older adults with low and high PNSC (χ^2^ = 34.27, *p* < 0.001). As depicted in Figure 1, the proportion of disability increased from 6.69% in older adults with robust status and high PNSC to 67% in older adults with frailty and low PNSC (χ^2^ = 246.89, *p* < 0.001).

### 3.4. Independent Impact of Physical Frailty and Perceived Social Cohesion on Functional Disability

Logistic regressions were sought to assess the independent associations of PF and PNSC with functional disability (Table 2 and Table S1). In the unadjusted model, both pre-frailty and frailty were significantly associated with functional disability (*p* < 0.001). In the adjusted model controlling for demographics, medical conditions and psychological factors, pre-frail older adults have over twofold increased risk (OR = 2.30, 95%CI = 1.49–3.55) of functional disability and frail older adults were over five times more likely (OR = 5.33, 95%CI = 3.17–8.96) to have functional disability, compared with robust older adults.

Our estimates from the logistic regressions also showed that low (vs. high) PNSC was significantly associated with increased risk of functional disability. In the unadjusted model, the older adults with low PNSC had a two-fold risk of functional disability (OR = 2.00, 95% CI = 1.56–2.57) compared with those with high PNSC. After adjusting for demographics, medical conditions and psychological factors, this association was attenuated slightly but remained significant (OR = 1.71, 95%CI = 1.28–2.27).

### 3.5. Combined Impact of Physical Frailty and Perceived Neighborhood Social Cohesion on Functional Disability

**Figure 2:**
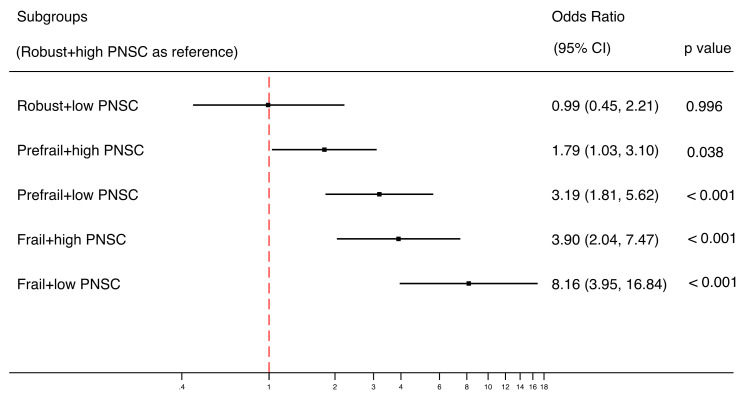
Combined relationships of PF and PNSC on functional disability using logistic model (*n* = 1616). A forest plot was used to display the estimated odds ratios (exponential form) for each group compared with the reference group (group of robust older adults with high PNSC), not comparing groups with each other. The analysis was adjusted for demographic, medical and psychological covariates.

The results of the associations between subgroups of PF with low or high PNSC are shown in Figure 2. Compared to the robust older adults with high PNSC, the pre-frail, high PNSC group was associated with higher likelihoods of functional disability (OR = 1.79, 95%CI = 1.03–3.10) and the pre-frail, low PNSC group was associated with over threefold increased risk of functional disability (OR = 3.19, 95%CI = 1.81–5.62). Frailty with high PNSC (OR = 3.90, 95% CI = 2.04–7.47) was associated with an approximate fourfold increased likelihood of functional disability compared to the robust group with high PNSC, whereas frailty with low PNSC stood out with over eightfold increased likelihood of functional disability (OR = 8.16, 95%CI = 3.95–16.84).

## 4. Discussion

The findings in this study contribute to our understanding of the independent and combined relationships of PF and PNSC on functional disability in community-dwelling older adults. As hypothesized, our study found that older adults with low PNSC were more likely to be have functional disability compared with older adults with high PNSC. Our estimates also show a tendency of increase in the odds of functional disability between low and high PNSC groups within physical frailty categories. However, in this study we were not able to detect a significant statistical difference between different PNSC groups with the same physical frailty category.

Previous studies have demonstrated that neighborhood social cohesion is associated with a range of health outcomes such as self-rated health, health-related quality of life, life satisfaction and use of preventive health services [19,30,40,41]. The findings in the current study expand the knowledge of the association between PNSC and functional disability. The results show that high PNSC based on friendship, visiting, borrowing and exchange of favors with neighbors was associated with maintaining functional ability in community-dwelling older adults. This relationship persisted even after adjusting for a series of demographic, medical and psychological covariates associated with functional disability. Considering that social cohesion is a component of cognitive social capital recognized by some researchers [24], this finding aligns with the results of previous studies reporting that low levels of social capital were associated with increased incidence of functional disability [42,43]. Given that older adults usually spend more time in their neighborhoods than younger adults [44], this association between PNSC and functional disability found in this study suggests that fostering social cohesion in the neighborhoods is a potential strategy to facilitate functional capacity and decrease the risk of disability in the older adults. Longitudinal studies are needed in the future to determine the direction in the association between PNSC and disability.

There are several possible explanations for the association between PNSC and functional disability. First, older adults with high PNSC may be more likely to seek social support or neighborhood assistance [45] when they experience a medical emergency (e.g., stroke, or myocardial infarction) so that they can have timely access to healthcare, which may eventually prevent the development of functional disability. Second, PNSC may decrease the risk of functional disability via social networking and group activities that promote health behaviors such as physical activity, healthy diet habits and smoking cessation [46,47,48]. Third, higher PNSC may contribute to mental health, which is associated with physical wellbeing, and thus eventually reduce the risk of functional disability. Previous research has shown that high PNSC is associated with lower negative emotion and fewer psychological distresses [27,49]. Given the research design of this study, though less likely, the lower PNSC might be attributed to restrictions in social activities due to functional disability. Longitudinal studies are needed in future to examine the causal relationship between PNSC and functional disability.

Despite the lack of statistical significance between low and high PNSC groups within physical frailty categories, differences in the estimates of odds ratios between those groups (e.g., odds ratio of 8.16 in the frail and low PNSC group vs. odds ratio of 3.90 in the frail and high PNSC group) implies older adults that are more frail and with lower PNSC are more vulnerable to having functional disability. One possibility of non-significant interaction between PNSC and physical frailty may be that we excluded the older adults who were unable to complete the physical performance tests of frailty. This underrepresentation of older adults who were severely frail might lead to an underestimation of the associations between physical frailty, PNSC and disabilities. Future research should consider conducting longitudinal studies with large sample size to determine the role of PNSC in the association between physical frailty and disability.

Our finding of the associations aforementioned has some implications. Health providers, especially those working in primary care settings and/or community care centers, should perform holistic assessment beyond physical assessment and include social determinants of health such as neighborhood social cohesion in order to provide patient-centered care. A growing body of research is demonstrating the critical roles social determinants of health play and has urged the inclusion of those factors into decision-making in health care planning and delivery [20,50,51]. This finding is of particular importance and has tremendous policy implications as the Chinese society is undergoing tremendous reconstruction in neighborhoods resulting from modernization and economic development. Future research should also investigate the impact of other neighborhood characteristics (e.g., neighborhood safety, sense of belonging) on frailty-associated functional disability and other health outcomes among older adults.

Not surprisingly, our study reported a strong association between frailty and functional disability, which is consistent with previous research [10,14,15]. Moreover, our finding on the relationship between the early stage of frailty, known as pre-frailty, and functional disability provides some new insights for preventing functional disability. Pre-frail older adults are more likely to transition back to being robust or remain stable than those who are frail [52]. Prefrailty is a stage that has a better likelihood of benefiting from intervention for reducing frailty and thus preventing disability [53]. This result highlights the importance of identifying prefrailty in community-dwelling older adults to detect early decline in functional capability.

Our findings also provide additional evidence demonstrating the high rate of functional disability in older adults in China, especially those living in urban areas in China. We found nearly one in four of the community-dwelling older adults aged 75 years or older in Shanghai, China had functional disability. This finding is consistent with reports by other researchers using similar measurements of functional disability. Ma et al. [37] reported the prevalence of functional disability was 25.1% among older adults aged 80 years or older in a national representative sample from seven provinces in China and Su et al. [54] found the rate of disability was 23.23% in ADL and 37.9% in IADL among community-dwelling older adults aged 80 years or older in Shanghai. Our findings, and results from previous research, highlight the urgency of addressing functional disability among older adults in China.

Several limitations of this study should be mentioned. First, this study used a cross-sectional design. We therefore were not able to identify the causality between PNSC, PF, and functional disability. Second, instead of operationalizing social cohesion as a community-level factor, we measured it at the individual level with reports from participants. Future studies may include a larger sample consisting of participants from various communities to examine the impact of social cohesion at an aggregated level as well as an individual level. Third, we excluded the older adults from the parent study if they did not complete the physical performance tests of physical frailty, which might result in a selection bias of underrepresenting older adults who were severely frail. Consequently, our findings might have underestimated the association between physical frailty and disabilities. Fourth, this study was conducted in Shanghai, China and thus our data is not nationally representative. We should be cautious in generalizing the conclusions of this study to other rural places in China. Last but not least, some unmeasured confounders (e.g., social participation [55]) may affect the associations between PF, PNSC and functional disability. Prospective studies investigating the links between other social determinants and functional disability may be worth conducting in future.

## 5. Conclusions

Findings from this study demonstrated the importance of PNSC in the risk of functional disability in community-dwelling older adults in China. The findings in this study have clinical implications. For health care providers, assessment and evaluation of social and neighborhood characteristics in the view of holistic care may be essential for frail older adults living in communities. For health policy makers, building cohesive and harmonious neighborhoods may be a potential strategy for preventing declines in functional capacity in older adults. Longitudinal studies conducted in diverse regions with different neighborhood features are needed in future to clarify the role of social cohesion on the development of functional disability or other adverse health outcomes in frail older adults.

## Figures and Tables

**Table 1:** Participants’ characteristics by status of functional disability (*n* = 1616).

		Total *n* (%)	Normal *n* (%)	Disability *n* (%)	*p* Value ^†^
Gender	Male	666 (41.3)	513 (41.7)	153 (40.1)	0.558
	Female	945 (58.7)	716 (58.3)	229 (59.9)	-
Age	75–80 years	811 (50.2)	718 (58.3)	93 (24.2)	<0.001 ^**^
	81–85 years	453(28.0)	333 (27.0)	120 (31.3)	-
	86 years and older	352 (21.8)	181 (14.7)	171 (44.5)	-
Educated Years	≤9 years	794 (49.5)	575 (47.0)	219 (57.3)	<0.001 ^**^
	>9 years	811 (50.5)	648 (53.0)	163 (42.7)	-
Marriage Status	Married	1060 (65.7)	846 (68.7)	214 (56.0)	<0.001 ^**^
	Unmarried	553 (34.3)	385 (31.3)	168 (44.0)	-
Family Income Monthly	<5000 Yuan	1070 (66.3)	800 (65.0)	270 (70.5)	0.046 ^*^
	≥5000 Yuan	544 (33.7)	431 (35.0)	113 (29.5)	-
Hypertension	Yes	1062 (65.7)	785 (63.7)	277 (72.1)	0.002 ^**^
Stroke	Yes	107 (6.63)	65 (5.28)	42 (11.0)	<0.001 ^**^
Heart Diseases	Yes	631 (39.1)	438 (35.6)	193 (50.3)	<0.001 ^**^
Diabetes Mellitus	Yes	357 (22.1)	268 (21.8)	89 (23.2)	0.557
Urine Incontinence	Yes	342 (21.3)	216 (17.7)	126 (33.0)	<0.001 ^**^
Chronic Pain	Yes	788 (48.8)	553 (45.0)	235 (61.2)	<0.001 ^**^
Visional Impairment	Yes	1257 (77.8)	941 (76.4)	316 (82.3)	0.015 ^*^
Hearing Impairment	Yes	887 (55)	629 (51.1)	258 (67.4)	<0.001 ^**^
Polypharmacy	≥5 Medications	155 (9.6)	105 (8.5)	50 (13.1)	0.008 ^**^
Hospitalization in Past Year	Yes	270 (16.7)	165 (13.4)	105 (27.3)	<0.001 ^**^
Falls in Past Year	Yes	169 (10.5)	115 (9.4)	54 (14.1)	0.008 ^**^
Cognitive Function	Intact	1043 (65.9)	866 (71.4)	177 (47.8)	<0.001 ^**^
	Impaired	540 (34.1)	347 (28.6)	193 (52.2)	-
Depression	Normal	1250 (78.4)	1017 (83.6)	233 (61.6)	<0.001 ^**^
	Depressed	345 (21.6)	200 (16.4)	145 (38.4)	-

Note: * *p* < 0.05; ** *p* < 0.01; † Chi-squared test.

**Table 2:** Independent associations of physical frailty and perceived social cohesion with functional disability (*n* = 1616).

		Unadjusted Model		Adjusted Model ^†^	
		OR (95% CI)	*p*	OR (95% CI)	*p*
PF	Robust	1.00		1.00	
	Pre-frail	3.95 (2.68, 5.82)	<0.001	2.30 (1.49, 3.55)	<0.001
	Frail	17.14 (11.02, 26,68)	<0.001	5.33 (3.17, 8.96)	<0.001
PNSC	High Level	1.00		1.00	
	Low Level	2.00 (1.56, 2.57)	<0.001	1.71 (1.28, 2.27)	<0.001

Note: OR = odds ratio; 95%CI = 95% confidence interval; PF = physical frailty; PNSC = perceived neighborhood social cohesion; † Adjusted for demographic, medical and psychological covariates, including age, gender, education level, marital status, family monthly income, medical history of hypertension, stroke, heart disease and diabetes mellitus, polypharmacy, visional impairment, hearing impairment, urine incontinence, chronic pain, history of hospitalization, fall history, depression, and cognitive impairment.

## Supplementary Materials

The following are available online at https://www.mdpi.com/1660-4601/17/16/5912/s1. Table S1: Associations with functional disability in logistic model.
