# Supplementary material for: Independent and Combined Relationships of Perceived Neighborhood Social Cohesion and Physical Frailty on Functional Disability in Community-Dwelling Older Adults
Source: Int J Environ Res Public Health. 2020 Aug 14;17(16):5912. doi: 10.3390/ijerph17165912 (PMC7460244; doi:10.3390/ijerph17165912)
Supplement: Supplementary file 1 [file ijerph-17-05912-s001.pdf]

Supplemental Table: Associations with functional disability in logistic model			
Variables		OR (95% CI)	p
Frailty	Robust	1	
	Pre-frail	2.30 (1.49,3.55)	<.001
	Frail	5.33 (3.17, 8.96)	<.001
PSC	High level		
	Low level	1.71 (1.28, 2.27)	<.001
Age		1.16 (1.12, 1.19)	<.001
Female		0.96 (0.69, 1.32)	0.787
Educated >9 years		1.16 (0.84,1.61)	0.371
Marital status	Married	1	
	Unmarried	1.20 (0.87, 1.66)	0.266
Income monthly	<5000 Yuan	1	
	>=5000 Yuan	0.93 (0.66,1.31)	0.677
Hypertension		1.18 (0.87, 1.62)	0.293
Stroke		1.76 (1.06, 2.92)	0.028
Heart diseases		1.38 (1.03, 1.86)	0.033
Diabetes mellitus		0.80 (0.57, 1.13)	0.213
Urine incontinence		1.35 (0.97, 1.88)	0.073
Chronic pain		1.38 (1.02, 1.86)	0.009
Visional impairment		0.84 (0.58, 1.22)	0.356
Hearing impairment		1.51 (1.11, 2.05)	0.009
Polypharmacy		0.92 (0.57, 1.47)	0.727
Hospitalization in past year		1.83 (1.29, 2.60)	0.001
Falls in past year		1.07 (0.70,1.65)	0.748
Cognitive impairment		1.59 (1.19, 2.11)	0.002
Depression		2.07 (1.50, 2.85)	<.001
